# Characterization of *Phlebotomus papatasi* Peritrophins, and the Role of PpPer1 in *Leishmania major* Survival in its Natural Vector

**DOI:** 10.1371/journal.pntd.0002132

**Published:** 2013-03-14

**Authors:** Iliano V. Coutinho-Abreu, Narinder K. Sharma, Maricela Robles-Murguia, Marcelo Ramalho-Ortigao

**Affiliations:** Department of Entomology, Kansas State University, Manhattan, Kansas, United States of America; Fundaçao Oswaldo Cruz, Brazil

## Abstract

The peritrophic matrix (PM) plays a key role in compartmentalization of the blood meal and as barrier to pathogens in many disease vectors. To establish an infection in sand flies, *Leishmania* must escape from the endoperitrophic space to prevent excretion with remnants of the blood meal digestion. In spite of the role played regarding *Leishmania* survival, little is known about sand fly PM molecular components and structural organization. We characterized three peritrophins (PpPer1, PpPer2, and PpPer3) from *Phlebotomus papatasi*. PpPer1 and PpPer2 display, respectively, four and one chitin-binding domains (CBDs). PpPer3 on the other hand has two CBDs, one mucin-like domain, and a putative domain with hallmarks of a CBD, but with changes in key amino acids. Temporal and spatial expression analyses show that *PpPer1* is expressed specifically in the female midgut after blood feeding. *PpPer2* and *PpPer3* mRNAs were constitutively expressed in midgut and hindgut, with *PpPer3* also being expressed in Malpighian tubules. *PpPer2* was the only gene expressed in developmental stages. Interestingly, *PpPer1* and *PpPer3* expression are regulated by *Le. major* infection. Recombinant PpPer1, PpPer2 and PpPer3 were obtained and shown to display similar biochemical profiles as the native; we also show that PpPer1 and PpPer2 are able to bind chitin. Knockdown of *PpPer1* led to a 44% reduction in protein, which in spite of producing an effect on the percentage of infected sand flies, resulted in a 39% increase of parasite load at 48 h. Our data suggest that PpPer1 is a component for the *P. papatasi* PM and likely involved in the PM role as barrier against *Le. major* infection.

## Author Summary

For a successful development within the midgut of the sand fly vector, *Leishmania* must overcome several barriers imposed by the vector that include the digestive proteases secreted within the midgut following a blood meal by the insect, the need to escape from the endoperitrophic space, and attachment to the midgut epithelia to prevent excretion with the remnants of the blood meal. The sand fly peritrophic matrix (PM) constitutes an important barrier against the establishment of *Leishmania* within the sand fly and if trapped within the PM these parasites will be passed along with the remnants of the blood meal. Despite the role of sand fly PM on *Leishmania* development, characterization of its molecular components and assessment of their roles against *Leishmania* are lacking. Thereby, we performed the molecular characterization of three *P. papatasi* peritrophins named PpPer1, PpPer2, and PpPer3. Overall, we demonstrated that: (1) PpPer3 displays a putative CBD domain that might have undertaken neo-functionalization, (2) *PpPer1* and *PpPer3* genes display differential gene expression upon *Le. major* infection; and (3) PpPer1 seems to be an important component for the function of *P. papatasi* PM as a barrier against *Le. major* infection.

## Introduction

Leishmaniasis is a neglected vector-borne disease caused by several different species of *Leishmania* [1], [2]. Current estimates of the distribution of leishmaniasis worldwide [3] indicate that endemic transmission occurs in 98 countries, with an approximate incidence of 500,000 new human cases are diagnosed annually, and 350 million people are at risk of becoming infected [4]. The DALY (or disability adjusted life years) burden for leishmaniasis is 2 million [4].

*Leishmania* are digenetic parasites, developing in a suitable mammalian host and within the sand fly vector [5]. To date, over 90 species of phlebotomine sand flies have been proven or incriminated as vectors of *Leishmania* [6].

In order to survive and successfully establish an infection in the sand fly, *Leishmania* must overcome many barriers (reviewed by [2]). First, and following ingestion with the blood meal, transitional stage *Leishmania* amastigotes must survive a proteolytic attack by digestive enzymes [7]–[10]. Upon developing into the promastigote stage, parasites (nectomonads) escape from the endoperitrophic space after PM breakdown [7], [11] and attach to the midgut epithelia [12], [13], in both cases to prevent excretion following the digestion of the blood meal. It has also been shown that an anterior plug prevents premature migration of nectomonads to anterior midgut [10]. As parasites develop into metacyclic promastigotes, they must detach from the midgut and migrate towards the foregut and the cardia (or stomodeal valve area). At the cardia, it has been shown that *Leishmania*-secreted chitinase damages the stomodeal valve preventing its normal function, and forcing the sand fly to regurgitate the contents of the gut as it attempts to blood feed [14], [15]. It is widely accepted that regurgitation carries *Leishmania* onto the skin of the vertebrate host, and this is the principal mechanism of parasite transmission.

Regarding the sand fly PM, earlier findings suggested that it serves as a barrier against *Leishmania* development [16], [17]. These results were further supported by feeding the chitinase inhibitor allosamidin to *P. papatasi* and showing that *Leishmania major* remained trapped inside a thicker PM [7]. These latter studies also revealed a dual role for the sand fly PM in protecting as well as serving as barrier to *Leishmania*. Altogether, these findings demonstrate that the PM is an important component of sand fly vector competence.

Despite its importance, little is known about the molecular components of the sand fly PM [18], [19], or their roles during infection with *Leishmania*.

Here, we characterized three peritrophins, PpPer1, PpPer2, and PpPer3, previously identified in the midgut of *P. papatasi* [18]. PpPer1 and PpPer2 are likely involved in the formation of the PM scaffold, as suggested by their expression profiles and ability of the respective recombinant proteins to bind exogenous chitin. PpPer3 on the other hand may be involved in mechanisms related to protection of the epithelia, as this peritrophin displays a mucin domain and is expressed in both gut tissues and Malpighian tubules. We also investigated the role of the sand fly PM as a barrier for *Leishmania* development. Our results indicate that reduction of PpPer1 expression levels leads to an increase in *Le. major* load in *P. papatasi*. Altogether, our results suggest that PpPer1 plays a significant role in *Le. major* development within the vector midgut.

## Materials and Methods

### Ethics statement

This study was carried out in strict accordance with the recommendations in the Guide for the Care and Use of Laboratory Animals of the National Institutes of Health. The use of animals in this study was reviewed and approved by the Committee on Institutional Animal Care and Use of the Kansas State University (KSU-IACUC) (Permit Numbers 2747, 2748 and 2749). All sand fly feedings on animals and all bleeds were performed on animals under anesthesia, and all efforts were made to minimize suffering.

### Bioinformatics analyses

The cDNA sequences of *PpPer1*, *PpPer2*, and *PpPer3* were previously identified in [18]. Predicted isoelectric points and molecular weights of mature proteins were obtained using the Compute pI/Mw tool [20]. Putative secretory signal peptides were determined using SignalP 3.0 [21]. Prediction of *O*-linked glycosylated amino acids was carried out with NetOGlyc 3.1 [22] while *N*-linked glycosylation site prediction was performed using NetNGlyc 1.0 (http://www.cbs.dtu.dk/services/NetNGlyc/). Protein domains were identified by searching Prosite (http://expasy.org/tools/scanprosite/), Pfam (http://pfam.sanger.ac.uk/search), and CDD (http://www.ncbi.nlm.nih.gov/Structure/cdd/cdd.shtml) domain databases. Chitin binding domain (CBD) classification in type-A (CX_13–20_CX_5–6_CX_9–19_CX_10–14_CX_4–14_C), type-B (CX_12–13_CX_20–21_CX_10_CX_12_CX_2_CX_8_CX_7–12_C), or type-C (CX_8–9_CX_17–21_CX_10–11_CX_12–13_CX_11_C) was performed visually, following the Consensus sequences described by Tellam [23]. The mucin-like domain amino acid composition was assessed using the GeneRunner software (http://www.generunner.net/). Predicted heme-regulatory motifs (HRM) were visually identified as cysteine-proline dipeptide [24].

Multiple sequence alignment of peritrophin CBDs was performed with the ClustalW tool in the BioEdit package [25]. Alignment was adjusted manually to remove some gaps. The CBDs of *P. papatasi* peritrophins were aligned to CBD sequences identified in peritrophins from *Lutzomyia longipalpis* [19], [26]. Alignment was performed with each CBD sequence located between the first and sixth conserved cysteine residues. The *L. longipalpis* peritrophin cDNA sequence identified in whole body libraries (NSFM-72d06.q1k; [26]), referred to here as LlPer3 is an ortholog of the *P. papatasi* PpPer3. A putative CBD was identified in the PpPer3 N-terminal sequence by visual inspection and named Pp3put. Ll3put was similarly identified within the *L. longipalpis* LlPer3. Peritrophin sequences displaying similarities to Pp3put and Ll3put CBDs were retrieved from GenBank and aligned to the sand fly CBDs.

Phylogenetic analysis was performed using the Maximum Likelihood method based on the Whelan and Goldman model [27]. The branch robustness was inferred by 500 bootstrap pseudo-replicates [28]. These analyses were carried out with the MEGA5 software [29].

### Sand fly samples, RNA isolation and cDNA synthesis

All sand flies used were *P. papatasi* (PPIS strain) reared at Department of Entomology, Kansas State University, as previously described [30]. For the adult flies 3-to-5 day-old insects were used in the experiments described below.

Blood feeding of sand flies was performed using two methods: 1) direct feeding on anesthetized (100 mg/kg of ketamine; and 3 mg/kg xylazine) BALB/c mice; and 2) using glass feeders filled with heat-inactivated mouse blood or heat-inactivated mouse blood mixed with 5×10^6^ *Le. major* amastigotes/ml [30]. Flies that were fed directly on the anesthetized mouse were used in the RT-PCR assays. The feeding of flies using the glass feeders was for flies used in real time PCR analyses, and for the flies injected with dsRNA. Only fully engorged sand flies were used.

*Phlebotomus papatasi* dissections, RNA isolations, and cDNA syntheses were performed according to [30].

Total RNA obtained from various tissues from adult females were dissected and pooled as follows. For midguts, five tissues from sugar fed (0 h) were dissected and combined. Likewise, five blood fed midguts also were dissected and combined at each of the following time points: 6 h, 12 h, 24 h, 36 h, 48 h, 72 h, 96 h, 120 h, and 144 h post blood meal (PBM). Pools of adult carcasses, hindguts, heads plus salivary glands, ovaries, and Malpighian tubules were made from tissues obtained from single sand flies dissected at 0 h, 6 h, 12 h, 24 h, 36 h, 48 h, 72 h, 96 h, 120 h, and 144 h PBM. The pool of fat bodies was made from single flies dissected at 6 h, 12 h, 24 h, and 36 h PBM. RNAs from developmental stages were obtained from pools of 20 eggs, 10 L1 larvae, and five each for stages L2, L3, L4, and five pupae. cDNAs obtained from RNA samples from pooled tissues were used in RT-PCR reactions described below.

For the real-time PCR (qRT-PCR) assays, *P. papatasi* midguts were dissected from flies that fed either on blood or blood plus *Le. major* (glass feeders) at 24 h, 48 h, and 72 h PBM. cDNAs obtained from eight RNA samples representing individual midguts from each of the three time points were used for real time PCR reactions below.

### RT-PCR and real time qRT-PCR

*PpPer1*, *PpPer2*, *PpPer3*, and *β-tubulin* cDNAs were amplified using primer pairs described in Table 1. The expression profiles of such genes were obtained after 23 amplification cycles for *PpPer2*, 25 cycles for *PpPer1* and *PpPer3*, and 28 cycles for *β-tubulin*. Reactions were performed in 25 µl total volume, containing 12.5 µl GoTaq Master Mix (Promega, Madison, WI), 1 µl cDNA, 0.5 pmoles each primer, and 10.5 µl molecular grade water. Amplification reactions were done as follows: 94°C for 3 minutes (min); 23–28 cycles of 94°C for 30 seconds (sec), 57–58°C for 1 min, 72°C for 30 sec; and a final amplification step at 72°C for 10 min.

**Table 1:** Primers list.

Primer Name	Nucleotide Sequence (5′ to 3′)	Annealing Temperature
PpPER1T7i_2_F	TAATACGACTCACTATAGGGAGAATGAAGAACGTTGCAGTGAT	55°C/65°C
PpPER1T7i_2_R	TAATACGACTCACTATAGGGAGATTAGTGGTCGAAGCAGACTG	55°C/65°C
PpPer1_122F	CTCATGAAGAGTTTTGCATG	57°C
PpPer1_122R	GAAACCGTCTTCACAGCTC	57°C
PpPer2_168F	5TGCCTGGTTTTCCTGTTC	58°C
PpPer2_168R	TCCTTGCACGAAAGTTCCC	58°C
PpPer3_121F	ATCTGCCCAGGACCATTAC	58°C
PpPer3_121R	AGTCGACTGTAGCGCAATC	58°C
PpTub_148F	GCGATGACTCCTTCAACAC	57°C
PpTub_148R	GTGATCAATTGTTCGGGATG	57°C
Pp40S_S3_136F	GGACAGAAATCATCATCATG	57°C
Pp40S_S3_136R	CCTTTTCAGCGTACAGCTC	57°C
PpPer1Mat_717F	GCTCATGAAGAGTTTTGCATG	56°C
PpPer1Mat_717R	TTAGTGGTCGAAGCAGACTG	56°C
PpPer2Mat_219F	GCCAATGTTTCTTGCCCACC	56°C
PpPer2Mat_219R	CTATTTTTGTCCGCCTGGAG	56°C
PpPer3Mat_863F	GAAGAAGTAGTACCAGGAATTC	56°C
PpPer3Mat_863R	TCATTTTCCTTGGGAAGATTG	56°C
PpPer1R-His	TTAGTGGTGATGGTGATGGTGGTCGAAGCAGACTG	*
PpPer2R-His	CTAGTGGTGATGGTGATGATGTTTTTGTCCGCCTGG	*
PpPer3R-His	TCAGTGGTGATGGTGATGATGTTTTCCTTGGGAAGATTG	*

Primer name, sequence, and annealing temperatures for the primer pairs used in dsRNA synthesis and real time PCR analyses.

* Described in Materials and Methods.

Real time quantitative PCR reactions were performed with a MasterCycler Realplex4 Eppendorf Real-Time PCR (Hamburg, Germany) using BioRad SyBR green (BioRad, Hercules, CA). Reactions were set up as described [30]. Amplification conditions and primer pairs used (Table 1) were the same used in RT-PCR reactions, except that a total of 40 amplification steps were performed. As a housekeeping control, cDNA corresponding to the S3 protein of the 40S ribosomal subunit was amplified (Table 1).

### Temporal expression of native peritrophins from *P. papatasi*

We assessed the expression of PpPer1, PpPer2 and PpPer3 proteins present in midgut lysates. Pools containing five midguts dissected from non blood fed and from blood fed *P. papatasi* at 24, 48, 72, 96 h PBM were homogenized in 50 µl PBS. Each midgut lysate was boiled in SDS lysis (Invitrogen) buffer for 5 min and one midgut equivalent from each midgut pool was separated under non reducing conditions on 4–12% Bis-Tris NuPAGE gels (Invitrogen). Proteins were transferred to nitrocellulose and incubated for 16 h at 4°C with anti-PpPer1 and anti-PpPer3 antisera diluted 1∶100 in TBS-T. Blots were washed and incubated with anti-mouse conjugated to alkaline phosphatase (1∶10,000) for 1 h. Blots were developed using Western Blue (Promega).

### Recombinant peritrophin expression

Mature cDNAs sequences (without the signal peptide) corresponding to each of the three peritrophins PpPer1, PpPer2, and PpPer3 were PCR amplified from *P. papatasi* midgut using specific primer pair combinations (PpPer1Mat_717F/PpPer1R-His, PpPer2Mat_219F/PpPer2R-His, and PpPer3Mat_867F/PpPer3R-His, Table 1), with each of the reverse primers containing a 6×-His tag (Table 1). PCR amplifications were performed as follows: three cycles of 95°C for 3 min, 94°C for 1 min, and 68°C for 1 min; five cycles of 94°C for 1 min and 62°C for 1 min; and 25 cycles of 94°C for 1 min, 60°C for 1 min, and 72°C for 1 min. Each PCR product was subsequently cloned and purified as described [30]–[32].

Recombinant proteins were expressed using cells (FreeStyle CHO-S) and reagents obtained from Invitrogen, and according to the manufacturer protocols. Transfected cells were incubated at 37°C (with 8% CO_2_) under gentle shaking (125 rpm). Culture supernatants were collected after 72 h and concentrated using Centricon filters (Millipore) at 3 kDa (rPpPer2) or 10 kDa (rPpPer1 and rPpPer3) cutoffs. Recombinant rPpPer1 and rPpPer2 also were produced using HEK293 cells and purified as described elsewhere [33].

### Purification of expressed protein

The concentrated supernatants for rPpPer1 and rPpPer3 recombinant proteins were further purified by Ni-NTA HisTrap column (GE Healthcare, Piscataway, NJ). Supernatants were filtered through a 0.22 µm Millex syringe filter (Millipore), and dialyzed overnight in PBS at 4°C. After dialysis each supernatant was manually injected into a HisTrap column, previously equilibrated with binding buffer A (20 mM sodium phosphate buffer, 500 mM sodium chloride, 10 mM imidazole, pH 7.4), and fitted to a HP1100 series HPLC system. Washes and elution of recombinant proteins were carried out according to [33] Different fractions corresponding to each wash interval with exception of the first 35 minutes were collected and analyzed by Comassie-stained SDS-PAGE and Western blot.

For purification of rPpPer2, a gravity flow column was used. The concentrated supernatant obtained from FreeStyle CHO-S cells was filtered and washed 5 times in PBS using a 3 kDa cutoff Centricon filter (Millipore), and loaded onto 1 ml Ni-NTA column in a 5 ml syringe (BD Biosciences). The column was washed with 15 ml 20 mM sodium phosphate buffer-300 mM sodium chloride-20 mM imidazole, and eluted with 5 ml 20 mM sodium phosphate buffer-300 mM sodium chloride-300 mM imidazole. The eluted rPpPer2 was concentrated (1.5 µg/µl) and analyzed with Commassie-stained SDS-PAGE and Western blot.

### Gel filtration chromatography

Native (from *P. papatasi* midgut extract) and recombinant proteins were subjected to HPLC gel filtration chromatography. The *P. papatasi* midgut extract was prepared from 10 midguts dissected 48 h PBM, pooled and homogenized in 50 µl of PBS pH 7.4 with 0.01% TritonX-100 followed by centrifugation at 14,000×g. The supernatant was collected and the pellet was extracted twice by sonication in 50 µl of PBS pH 6.8, followed by centrifugation as above. The three resulting supernatants were combined. For the recombinant proteins, Ni-NTA purified rPpPer1, rPpPer2, and rPpPer3 were used.

A 7.8×300 mm Bio-Sil SEC-250 column (Biorad) was fitted onto a HP Agilent 1100 series HPLC (Hewlett Packard, Santa Clara, CA), and each protein was separately loaded onto the column. The HPLC was performed using PBS pH 6.8 at isocratic flow rate of 1 ml per minute. Absorbance of eluted was measured at 280 nm using the HP1100 series variable wavelength detector. One ml fractions of the HPLC elute (both from midgut lysate or recombinant proteins) were collected in 1.5 ml tubes and 10 µl of each fraction was blotted onto the nitrocellulose membrane (dot blot). Dot blot filters were incubated to corresponding anti-sera and to anti-His antibodies as indicated below (Western blot). Fractionation and dot blots were repeated twice for each recombinant protein and for the midgut lysate. Recombinants rPpPer1 and rPpPer2 obtained from HEK-293 cells also were fractionated using the HP1100 series HPLC, and collected fractions were applied to dot blots and incubated with anti-His or with specific antisera, as described above.

### *N*-linked deglycosylation of rPpPer2

*N*-linked deglycosylation was carried out with PNGase F (New England Biolabs Inc., Ipswich, MA). Briefly, 200 ng of purified rPpPer2 was incubated separately with 1000, 2000 and 3000 units of PNGase F overnight at 37°C in 20 µl reaction. The reaction was stopped by addition of SDS gel loading buffer (Invitrogen), the proteins were separated on 4–12% pre-cast Bis-Tris NuPAGE gel (Invitrogen), transferred to nitrocellulose, and analyzed by western blot.

### Chitin binding assays

We assessed the ability of the purified 6× His-tagged recombinant proteins to independently bind colloidal chitin according to [34]. Briefly, two micrograms of each recombinant protein was mixed with colloidal chitin suspended in 100 µl 10 mM sodium phosphate buffer, pH 8.0, and incubated at room temperature for 1 h followed by centrifugation at 10,000×g. The supernatant was saved as unbound protein and the chitin pellet was washed with 50 µl of the same buffer and centrifuged as indicated above (wash one or w1). Additional washes were performed with 10 mM sodium phosphate buffer containing 1 M sodium chloride, pH 8.0 (w2) and 0.1 M acetic acid (w3), respectively. The final pellet was boiled in 50 µl of SDS-PAGE sample buffer (Invitrogen), centrifuged, and the supernatant collected. Unbound protein, washes, and SDS eluted fractions were separated in reducing condition with 2% betamercaptoethanol (β-ME) on pre-cast 4–12% Bis-Tris NuPAGE (Invitrogen). Proteins were transferred to nitrocellulose and analyzed by Western blot.

### Antisera production and Western blot

Antisera production was performed as described previously [31], [32], [35]. The mature sequences for PpPer1, PpPer2, and PpPer3 were amplified from *P. papatasi* midguts (12 h PBM), using the primer pairs PpPer1Mat_717F/PpPer1Mat_717R, PpPer2Mat_219F/PpPer2Mat_219R, and PpPer3Mat_867F/PpPer3Mat_867R (Table 1), respectively. Amplifications were as follows: 94°C for 3 min; 35 cycles of 94°C for 1 min, 56°C for 1 min, and 72°C for 1 min; and final extension at 72°C for 10 min.

To determine the effects of dsRNA injection on the expression of PpPer1 in *P. papatasi* midguts, polyclonal anti-PpPer1 specific antisera (1∶50 dilution), as well as Western blot assays were performed according to [30]. Densitometry analysis was performed using the TotalLab TL100 software (Nonlinear Dynamics, Durham, NC).

Western blots also were performed for the analyses of the 6×His-tagged recombinant proteins rPpPer1, rPpPer2, and rPpPer3. The concentration of each recombinant protein was determined using the BCA Protein Assay Kit (Thermo Scientific, Rockford, IL) or by measuring OD at 280 nm. Recombinant proteins (purified or concentrated FreeStyle CHO-S supernatant) were fractionated on 4–12% reducing Bis-Tris NuPAGE pre-cast gels (Invitrogen). Proteins were transferred to a nitrocellulose filter (Whatman, Dassel, Germany), incubated overnight at 4°C with anti-His antibody (Santa Cruz, Santa Cruz, CA) diluted 1∶2,000 or with different antisera, followed by three washes of 10 minutes each in TBS-T (TBS buffer with 0.1% tween-20). Each blot was incubated with anti-mouse antibody conjugated to alkaline phosphatase (Promega) diluted 1∶10,000 in TBS-T for 1 h at room temperature and washed in TBS-T as indicated above. The protein bands were visualized using the Western Blue (Promega).

Alternatively, Western blots were incubated with anti-mouse-HRP conjugated secondary antibody (Promega) diluted 1∶10,000, and detected with SuperSignal West Pico Chemiluminescence Substrate (Thermo Scientific) in chemiluminescence assays.

### Knockdown of PpPer1

PpPer1 was selected for these studies in light of its mRNA expression profile (midgut-specific and regulated by blood feeding). Double-strand RNAs were synthesized using the Megascript RNAi kit (Ambion). Synthesis and purification of dsRNA as well as injections of sand flies were performed according to [30]. The dsRNA targeting PpPer1 (dsPpPer1) was PCR amplified with primers PpPER1T7i_2 forward and reverse (Table 1). dsGFP was used as control, as described [36]. The effects of dsRNA induced knockdown were assessed by real time PCR analyses and Western blot.

Next, we assessed the effects of PpPer1 knockdown on *Le. major* development within the *P. papatasi* midgut. In that case, 80.5 ng of dsRNA was injected intra-thoracically per sand fly [30]. After feeding on an infectious blood meal, midguts were individually dissected at 48 h and 96 h PBM and homogenized in 30 µl PBS (pH 7.4). Live parasites were counted using a hemocytometer.

### Statistical analyses

Unpaired t-test and Mann-Whitney U test were used to assess for statistically significant differences in expression profiles and parasite counts when data followed or not a normal distribution, respectively. Assessment of distribution pattern was carried out by D'Agostino & Pearson omnibus normality test. Differences were considered statistically significant at p<0.05. All the statistical assessments were performed using GraphPad Prism v.5.0 (GraphPad Software, Inc).

### Accession numbers

Sequence accession numbers: Sand flies. PpPer1 (Gen Bank accession number: EU031912). PpPer2 (EU047543). PpPer3 (EU045354). LuloPer1 (EU124588). LuloPer2 (EU124602). LuloPer3 (EU124607). LlPer3 (AM093395). *C. felis*. PL1 (AAM21354). *C. quinquefasciatus*. conserved hypothetical protein (XP_001864216). *A. aegypti* AaeL_AAEL012651 – Ae51put (XP_001662775), AaeL_AAEL012645 – Ae45put (XP_001662776), and AeputAaeL_AAEL012652 – Ae52put (XP_001662772).

## Results

### Peritrophins cDNA sequences and predicted protein organization

The complete cDNA sequences of *P. papatasi PpPer1*, *PpPer2*, and *PpPer3* were previously identified and published in [18].

**Figure 1:**
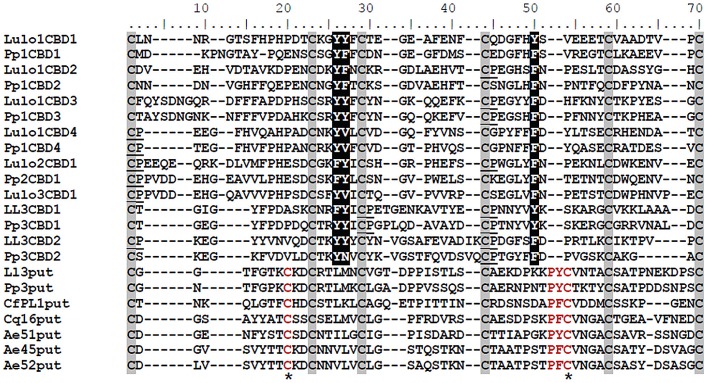
Multiple sequence alignment. Multiple sequence alignment was performed with individual CBD domains identified from peritrophins from *P. papatasi* and *L. longipalpis*. Pp1CBD1, Pp1CBD2, Pp1CBD3, and Pp1CBD4 are PpPer1 CBDs. Pp2CBD1 is the single CBD in PpPer2. Pp3CBD1 and Pp3CBD2 are the two CBDs identified in PpPer3. Lulo1CBD1, Lulo1CBD2, Lulo1CBD3, and Lulo1CBD4 are the four CBDs in LuloPer1. Lulo2CBD1 and Lulo3CBD1 are the single CBDs in LuloPer2 and in LuloPer3, respectively. Ll3CBD1 and Ll3CBD2 are CBDs in LlPer3. Pp3put and Ll3put are putative domains similar to CBDs identified in *P. papatasi* PpPer3 and in *L. longipalpis* Ll3Per3. Such putative domain sequences also were identified in the N-terminal region of *C. felis* PL1 - CfPl1put; *C. quinquefasciatus* conserved hypothetical protein - Cq16put; and *A. aegypti* Ae51put, Ae45put, and Ae52put. The six conserved cysteines are highlighted in grey with the conserved aromatic amino acids predicted to bind chitin shown in white with black highlight; HRM motifs are underlined. Conserved amino acid residues displayed exclusively by the putative CBD domain sequences are shown in red, and the additional cysteine residues are indicated by asterisk (*).

*PpPer1* open read frame (ORF) is 792 bp long, encoding a protein of 263 amino acids, with a predicted molecular weight of 28 kDa for the mature protein, and an acidic pI (4.84). Putative *N*- and *O*-linked glycosylation at residues N29 and T211, respectively, are expected to add to the molecular weight of the secreted protein. PpPer1 displays a predicted signal peptide (amino acid residues 1–18), suggesting the protein is secreted into the midgut lumen. Four type-A CBDs (PpPer1 CBD consensus sequence: CX_13–19_CX_5_CX_9–10_CX_12_CX_7_C) are also present in the mature protein. In addition, two putative HRM were identified at amino acid residues 182–183 in the third CBD (Pp1CBD3 residues 44 and 45 in Figure 1), and at residues 209–210 in the fourth CBD (Pp1CBD4 residues 1 and 2 in Figure 1).

*PpPer2* ORF is 270 bp coding for a predicted 7.8 kDa mature protein with a single type-A CBD (consensus sequence: CX_18_CX_5_CX_9_CX_12_CX_7_C). Predicted *N*-glycosylation at amino acid residues N19 and N77 are also expected to increase the molecular weight of the protein. PpPer2 is an acidic protein (pI 4.25), with a single putative HRM at residues 22 and 23 of the predicted CBD (corresponding to residues 1 and 2 of Pp2CBD1 in Figure 1). The presence of a signal peptide with cleavage site between amino acids A17 and A18 suggests the protein is secreted into the midgut lumen.

*PpPer3* is 313 bp with two CBDs. Unlike PpPer1 and PpPer2, PpPer3 has a mucin-like domain rich in serine (18.2%), threonine (36.4%), proline (12.1%), and glutamine (12.1%) residues in addition to two type-A CBDs (PpPer3 CBD consensus sequence: CX_11_CX_5_CX_11–13_CX_12_CX_4–8_C). The predicted molecular weight of the mature PpPer3 is 32 kDa. Moreover, this peritrophin has a neutral-to-basic pI (7.75). As a large number of residues (281T, 285T, 286T, 292S, 293T, 294T, 295T, 296T, 300S, 301S, 302S, 303T, 304T, 305T, 306T, 307T, 309S, 310S) within the mucin-like domain are predicted to be *O*-linked glycosylated, PpPer3 molecular mass is expected to be significantly greater. Two additional features of PpPer3 are the presence of a 57-residue long linker between the first (Pp3CBD1) and second (Pp3CBD2) CBDs, and an N-terminal sequence containing eight cysteine residues (Pp3put, in Figure 1). Although the Pp3put sequence displays a type-A CBD signature (CX_10_CX_5_CX_11_CX_14_CX_10_C) similar to other CBDs in *P. papatasi* peritrophins, it was not recognized as a *bona fide* CBD by standard bioinformatics' tools. Two predicted HRM were identified at residues 138–139 (corresponding to residues 29 and 30 in Pp3CBD1, Figure 1) and 150–151 (residues 44 and 45 in Pp3CBD1, Figure 1) in the first PpPer3 CBD while a single HRM was predicted in residues 262–263 (residues 44 and 45 in Pp3CBD2) in the second PpPer3 CBD sequence (Figure 1).

According to the multiple sequence alignment between *P. papatasi* and *L. longipalpis* peritrophin CBDs (Figure 1), the six conserved cysteine residues characteristic of type-A CBDs are present. Interestingly, the numbers of amino acid residues between the second and third cysteines, and between the fourth and fifth cysteines were the least variable. In addition, aromatic residues Y and F corresponding to positions 25 and 26 between the second and third cysteines, and position 48 between the fourth and fifth cysteines were detected. Regarding the HRM sites, most are co-localized with the first and fourth cysteine residues.

Pp3put, a putative CBD domain present in the N-terminal portion of *P. papatasi* PpPer3 peritrophin, displays two extra cysteine residues at positions 18 and 53, and two residue insertion (PY) between the fourth and fifth conserved cysteines (Figure 1). This putative CBD domain displays neither HRM motifs nor aromatic residues at positions 25, 26, and 48. Interestingly, other insect peritrophins with features similar to Pp3put were also identified by searching the GenBank database against this putative CBD domain from *P. papatasi*.

**Figure 2:**
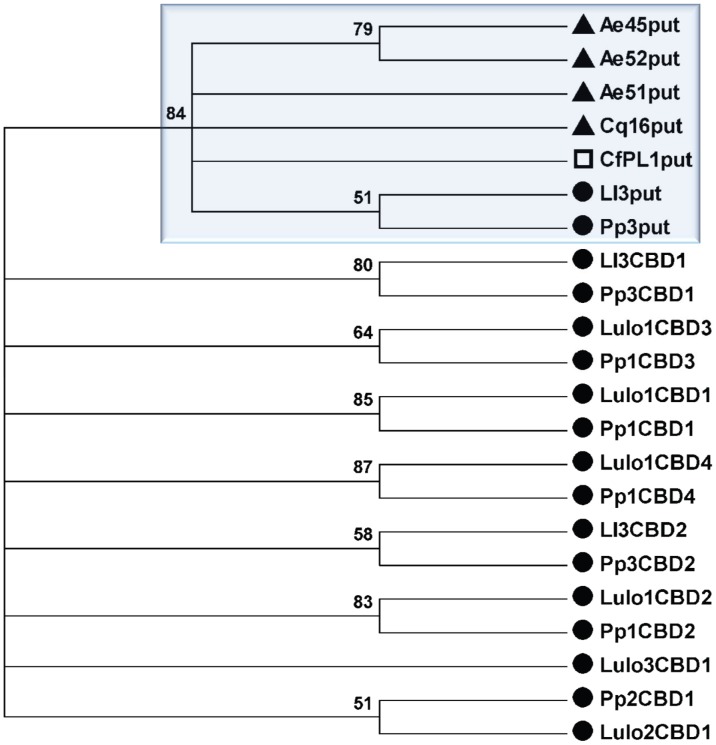
Phylogenetic comparison. Condensed tree depicts all the putative CBD domains in a single branch (blue shadow box), displaying strong bootstrap support (84%). All other branches are CBDs found in orthologs of sand fly peritrophins. Filled circles, filled triangles, and open square indicate sand fly, mosquito, and flea peritrophins, respectively.

A phylogenetic analysis suggests a single clade for the Pp3put and Ll3put domains from sand flies (*P. papatasi* and *L. longipalpis*), the Ae45put, Ae52put, and Ae51put from *Ae. aegypti*, the Cq16put from *C. quinquefasciatus*, and the CfPL1put from *C. felis* (Figure 2, blue box). The phylogenetic analysis also highlights the elevated conservancy that exists between the CBD domains in *P. papatasi* and *L. longipalpis* orthologous peritrophins, as reported previously [18]).

### Peritrophin mRNA expression profiles

**Figure 3:**
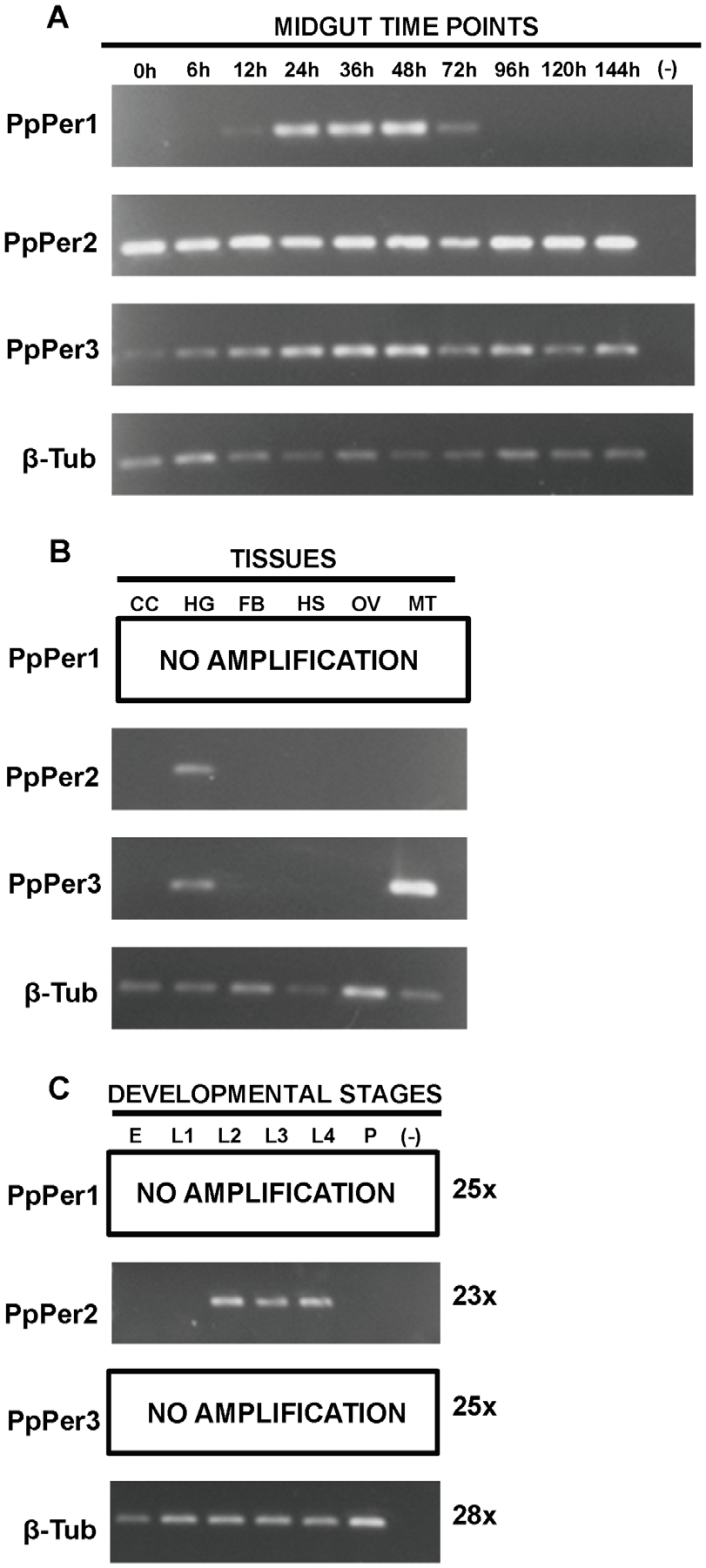
Peritrophin mRNA expression profiles. Expression of *PpPer1*, *PpPer2*, and *PpPer3* mRNAs was assessed by RT-PCR (23–25 cycles) in *P. papatasi* midguts dissected from adult females at different time points before and after blood feeding (0–144 hours PBM) (**A**); in pools of tissues other than midgut (**B**); and in eggs (pool of 20 eggs) or whole body of larvae and pupae (**C**). β-Tub was used as the housekeeping control gene. The size of the cDNA fragments amplified were 122 bp (*PpPer1*), 168 bp (*PpPer2*), 121 bp (*PpPer3*), and 148 bp (β-Tub). CC: Carcass. HG: Hindgut. FB: Fat Body. HS: Head along with salivary glands. OV: Ovaries. MT: Malpighian Tubules. E: Eggs. L1: Larval stage 1. L2: Larval stage 2. L3: Larval stage 3. L4: Larval stage 4. P: Pupa. (-): Negative control.

The expression profiles of *PpPer1*, *PpPer2*, and *PpPer3* were assessed by semi-quantitative RT-PCR (Figure 3). *PpPer1* mRNA expression was adult midgut-specific and blood-induced (Figure 3A and 3B); transcripts were detected between 12 h and 72 h PBM, with the highest levels at 48 h PBM. *PpPer2* transcripts were expressed in the midgut and in the hindgut (Figure 3A and 3B). *PpPer2* was constitutively expressed in sugar (0 h) and blood fed guts (Figure 3A). *PpPer3* was expressed in the midgut (Figure 3A) and in the hindgut and Malpighian tubules (Figure 3B). In spite of being expressed in sugar (0 h) and blood fed midguts, *PpPer3* mRNA expression was up-regulated between 12 h and 48 h PBM, somewhat similar to the *PpPer1* expression profile. Among the three *P. papatasi* peritrophins, only *PpPer2* was expressed in larval stages (Figure 3C) and, comparatively, also appeared to have the highest expression levels of the three peritrophins, according to Figure 3A.

### Temporal expression of native PpPer1 and PpPer3 in *P. papatasi*

**Figure 4:**
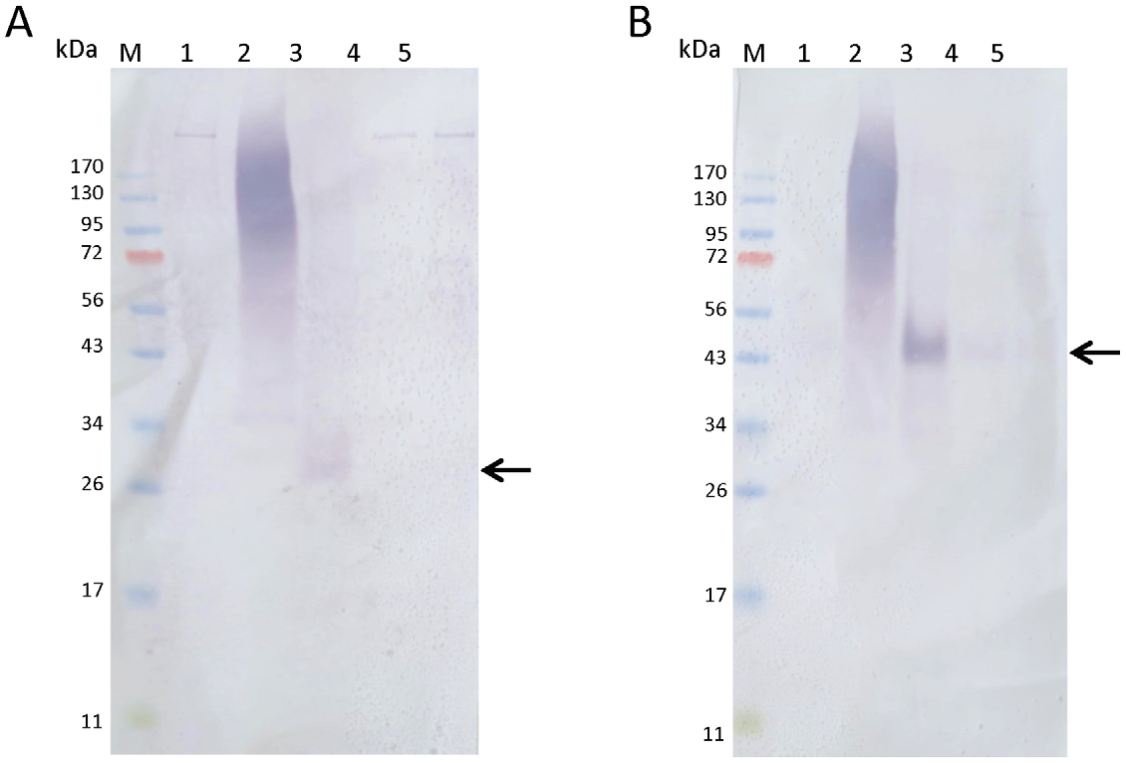
Expression of PpPer1 and PpPer3 in *P. papatasi* midgut lysates. Expression of native PpPer1 and PpPer3 in *P. papatasi* midgut lysates was assessed using one midgut equivalent (from pools of five guts for each time point) of non blood fed and blood fed *P. papatasi* per lane. Lanes: M, size marker; 1, non blood fed midgut; 2, blood fed midgut dissected 24 h PBM; 3, blood fed midgut dissected 48 h PBM; 4, blood fed midgut dissected 72 h PBM; 5, blood fed midgut dissected 96 h PBM. Proteins were transferred to nitrocellulose and blots were incubated with anti-PpPer1 (A) and with anti-PpPer3 (B) specific antisera. Arrows point the native proteins PpPer1 (A) and PpPer3 (B).

PpPer1 and PpPer3 are secreted in *P. papatasi* midgut following a blood meal, and are easily detected by Western blot at 48 h PBM (Figure 4). PpPer3 is also clearly present in midgut lysates dissected at 72 h and at 96 h PBM. Unfortunately, we were unable to resolve the smear visible in the lysates prepared from midguts dissected at 24 h PBM under non reducing conditions, and the specific antisera anti-PpPer1 and anti-PpPer3 did not bind to the native proteins under reducing conditions. The smear present in the 24 h samples is likely due to cross reaction of the antisera with mouse blood, and in our view irrelevant the findings. Nevertheless, at least for the native PpPer1, its expression profile is in accordance to the mRNA expression profile observed in Figure 3A.

### Expression of *PpPer1* and *PpPer3* is modulated by *Le. major*

**Figure 5:**
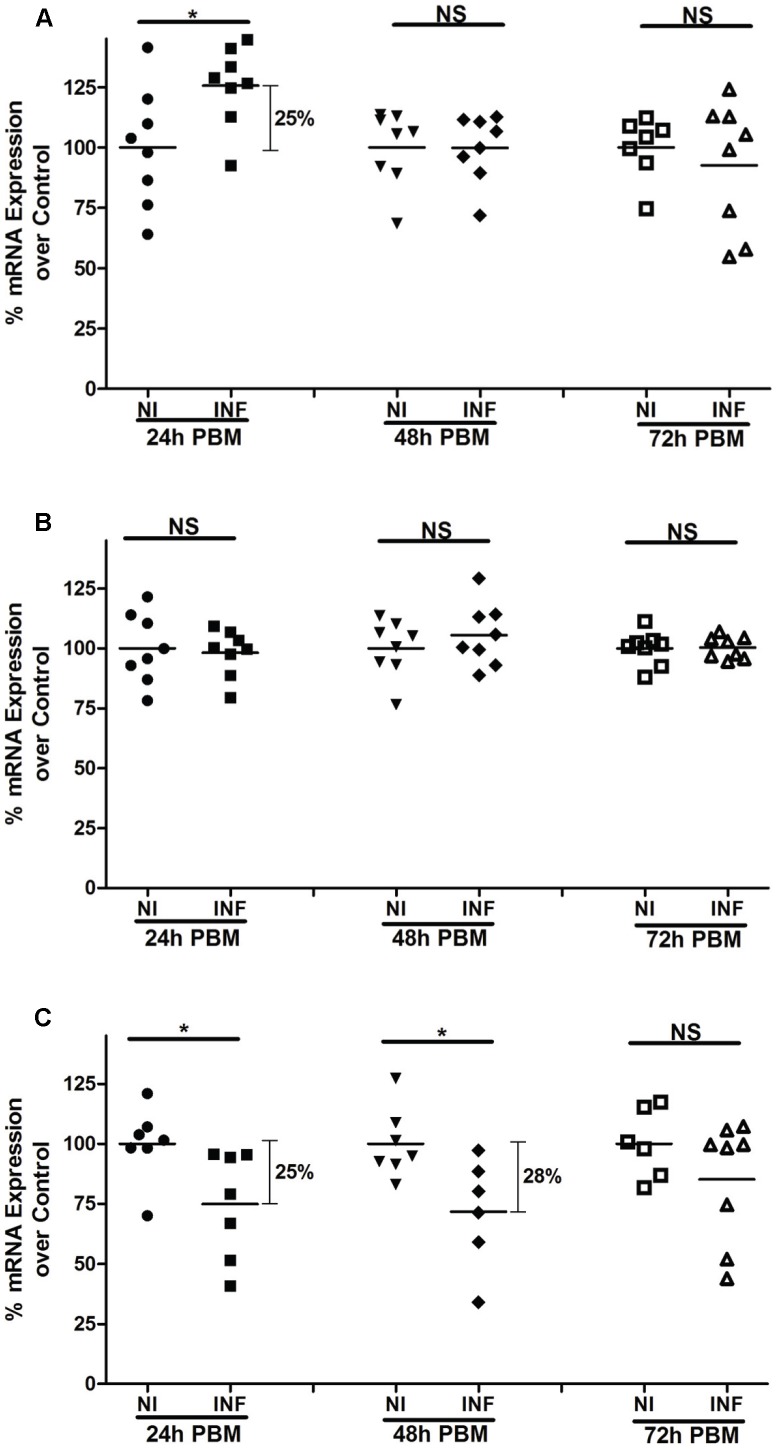
Modulation of peritrophin mRNA expression upon *Le. major* infection. qRT-PCR assays depicting differences in PpPer1 (**A**), PpPer2 (**B**), and PpPer3 (**C**) mRNA levels between non-infected and *Le. major* infected midguts dissected at 24 h, 48 h, and 72 h PBM. Each dot (symbol) represents the mRNA expression levels in a single midgut whereas horizontal bars indicate mean expression levels. The cDNA encoding the S3 protein of the 40S ribosomal subunit was used as the housekeeping control gene. The mean expression of non-infected midguts was used as a standard (100%) for comparisons to the percentage of mRNA expression of *Le. major* infected midguts for each time point. NI: Non-infected. INF: *Le. major* infected. NS: Not significant. *: Statistically significant, p<0.05.

We evaluated the effects of *Le. major* infection on expression of *P. papatasi* peritrophin mRNAs (Figure 5). *PpPer1* expression displayed a statistically significant up-regulation (20%) at 24 h post-infection (Figure 5A). However, no statistical difference was observed for *PpPer1* midgut expression at later time points (48 h and 72 h) following *Le. major* infection. No difference was observed for *PpPer2* for the three time points assessed (Figure 5B). For PpPer3, the mucin-like peritrophin, midgut mRNA levels were reduced by 25% at 24 h and 28% at 48 h after *Le. major* infection (Figure 5C). No differential *PpPer3* expression was observed in *Le. major* infected midguts at 72 h post-infection.

### Recombinant proteins

**Figure 6:**
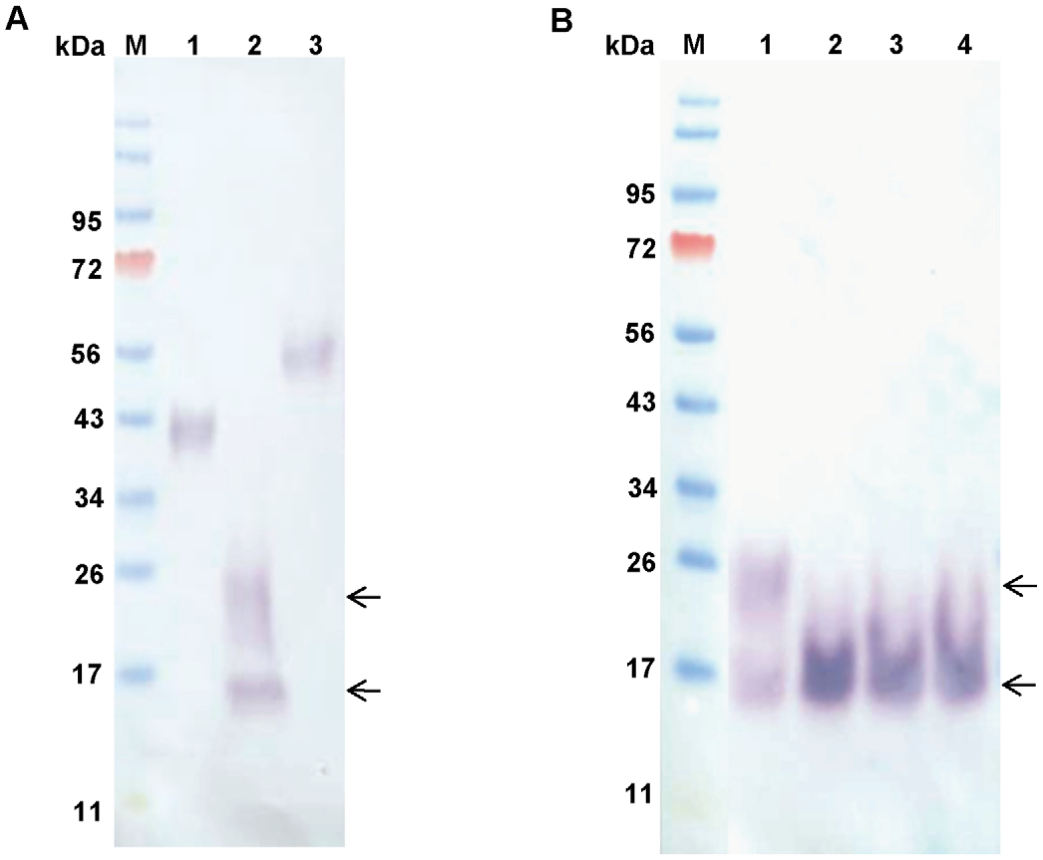
Recombinant proteins. Western blots were carried out with 6×His-tagged rPpPer1, rPpPer2, and rPpPer3 (250 ng each protein) obtained from FreeStyle CHO-S cells. Proteins were separated on 4–12% reducing NuPAGE gels. (**A**) Proteins were transferred to nitrocellulose and incubated with anti-His antibody (1∶2,000), followed by anti-mouse AP-conjugated (1∶10,000). Lanes: M, molecular weight marker; 1, PpPer1; 2, PpPer2; 3, PpPer3. (**B**) rPpPer2 (200 ng) was *N*-deglycosylated with 1,000 (lane 2), 2,000 (lane 3) and 3,000 (lane 4) units of PNGase F for 16 h at 37°C (untreated rPpPer2 control lane 1). Separated proteins were transferred to nitrocellulose and incubated with anti-His followed anti-mouse AP-conjugated. M: molecular weight marker. Arrows indicate the 16 kDa and 20-to-24 kDa bands seen on rPpPer2 preparations (**A** and **B**). After PNGase F treatment the 20-to-24 kDa bands are no longer detected.

Recombinant, 6×His-tagged, rPpPer1, rPpPer2, and rPpPer3 were successfully obtained using the FreeStyle CHO-S cells (Figure 6A). Posttranslational modifications (e.g., glycosylation) likely were responsible for the increased molecular weight detected for the recombinant proteins. Interestingly, mass spec analysis of rPpPer2 indicated a major peak at 9.2 kDa, with a minor peak at 18.4 kDa, suggestive of dimerization of this protein (not shown). In contrast, the Western blot showed the presence of bands at approximately 16 kDa and 20-to-24 kDa in rPpPer2 (Figure 6A). The higher molecular bands were no longer detected following digestion with PNGase F indicative of *N*-linked glycosylation (Figure 6B). Differences between the predicted (7.8 kDa) and the estimated sizes for rPpPer2 using mass spec and Western analyses might have been due to the presence of the 6×His tag interfering with gel migration, to non-predicted *O*-ring glycosylation(s), or to incomplete digestion of *N*-linked residues. Similar SDS-PAGE migration discrepancies were observed for the peritrophin 15 of the screwworm fly *Chrysomya bezziana* [37].

The recombinant proteins were analyzed by gel filtration column. The retention time for rPpPer1 matched that detected for the native molecule present in the *P. papatasi* midgut lysate. For PpPer1, elution from the midgut lysate occurred between fractions 6 and 7 (dot blot 1 in Supporting Figure S1A), while the rPpPer1 was eluted with fractions 6–8 (Supp. Figure S1B, dot blot). For rPpPer2, the protein was eluted mainly in fractions 11–13 (Supp. Figure S1C, dot blot). We were unable to generate polyclonal anti-sera to PpPer2 to efficiently detect fraction from the midgut lysate. Nevertheless, our data clearly demonstrates that the rPpPer2 was produced, as determined by hybridization with the anti-His antibody (Supp. Figure S1C). For PpPer3, while the recombinant protein had a wide trailing, being eluted with fractions 6–13 (Supp. Figure S1D) the native protein was eluted with fractions 6 and 7 (dot blot 2 in Supp. Figure S1A).

### Chitin binding assays

**Figure 7:**
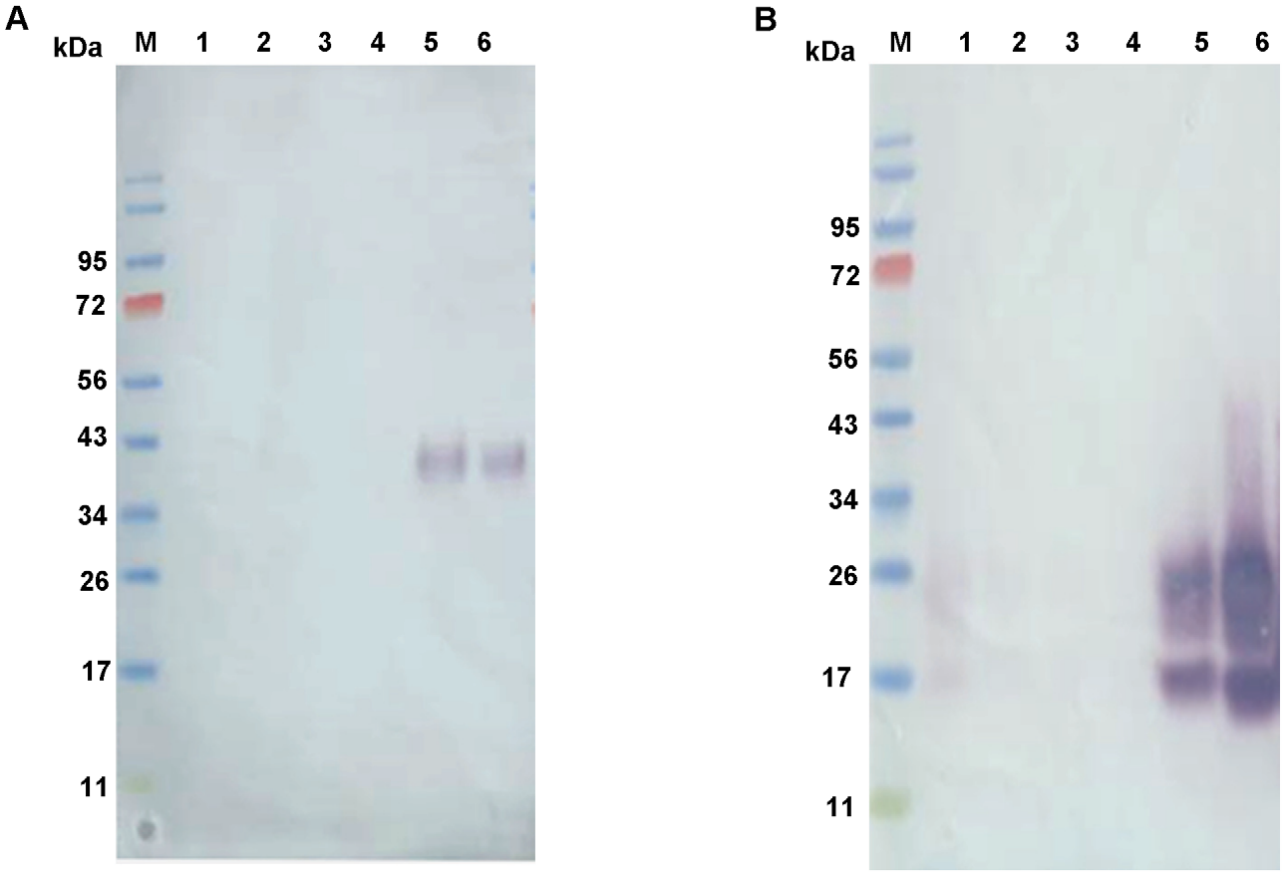
Chitin binding assay. Recombinant rPpPer1 and rPpPer2 were assayed for the capacity to bind colloidal chitin. Wash and elute fractions corresponding to PpPer1 (**A**) and rPpPer2 (**B**) were loaded onto reducing 4–12% NuPAGE gels. Lanes: M, molecular weight marker; 1, unbound fraction; 2, wash 1 (10 mM sodium phosphate buffer, pH 8.0); 3, wash 2 (10 mM sodium phosphate buffer, pH 8.0 - 1M sodium chloride); 4, wash 3 (0.1 M acetic acid); 5, elute (SDS lysis buffer); 6, purified protein. Proteins were transferred and blots were incubated with anti-His followed by anti-mouse AP-conjugated.

Figure 7 shows the results of the binding of the recombinant proteins to colloidal chitin. No recombinant protein in detected in the wash fractions obtained from the colloidal chitin binding assays (washes 1–3). Accordingly, rPpPer1 (Figure 7A) and rPpPer2 (Figure 7B) were only detected after boiling in the presence of SDS, thus demonstrating the ability of both rPpPer1 and rPpPer2 to bind chitin. In contrast, we were unable to demonstrate binding of rPpPer3 to chitin.

### *PpPer1* knockdown affects *Le. major* load within *P. papatasi*

*PpPer1* was selected for the knockdown experiments following our assessments of its expression profile (Figure 3) and according to the data from chitin binding assays. As PpPer1 is expressed exclusively in the midgut after blood feeding and binds chitin, we reasoned it was involved in PM formation.

**Figure 8:**
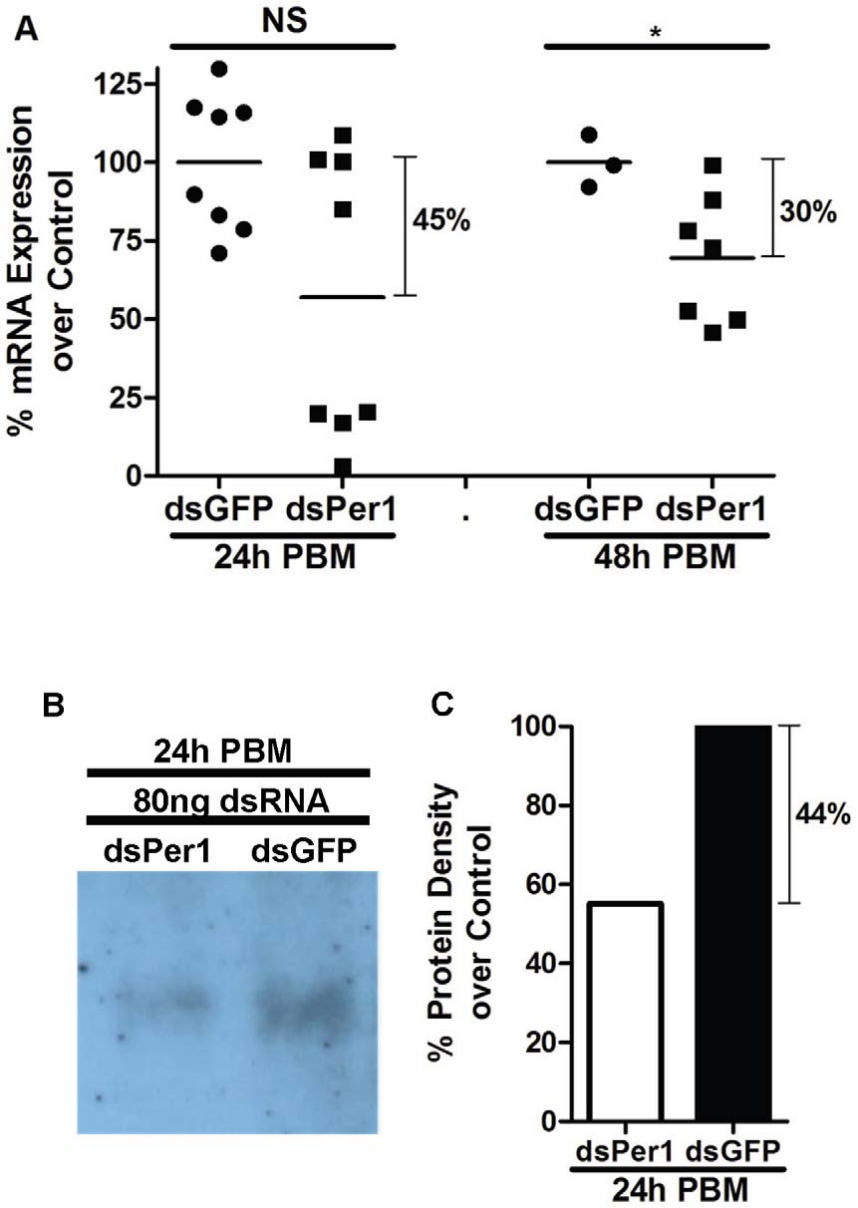
*PpPer1* knockdown at mRNA and protein levels. (**A**) real time qRT-PCR showing *PpPer1* mRNA level reduction in dsRNA-injected (dsPer1) *P. papatasi* compared to control injected (GFP dsRNA). Knockdown effects in *PpPer1* mRNA expression were assessed at 24 h and 48 h PBM that corresponded to 72 h and 96 h post dsRNA injection. Each symbol represents mRNA expression levels in a single midgut. Horizontal bars indicate mean expression levels. The S3 gene was used as the housekeeping control gene. The mean expression of *PpPer1* in dsGFP-injected flies was used as 100% standard. NS: Not significant. *: Statistically significant, p<0.05 (Unpaired t-test). (**B**) Western blot assay showing reduction in PpPer1 protein levels at 24 h PBM (72 h after dsRNA injection) in dsPer1-injected flies compared to dsGFP-injected (chemiluminescence development). Nine and a half micrograms of protein were loaded onto 10% NuPAGE gel. (**C**) Densitometry showing 44% reduction in the intensity of PpPer1 protein band obtained after chemiluminescence development compared to the PpPer1 band intensity of dsGFP-injected flies. For all *PpPer1* knockdown assays, 80 ng of dsRNA was injected intrathoracically into 3-to-5 day old *P. papatasi* females fed on 30% sucrose solution *ad libitum*.

Intra thoracic injections of *P. papatasi* females with 80.5 ng of double-strand RNA specific for *PpPer1* (dsPpPer1) were performed to assess the role, if any, of PpPer1 protein on *Le. major* development. First we determined whether injection of dsPer1 was able to reduce mRNA and protein levels. As shown in Figure 8, injection of the dsPpPer1 led to 45% and 30% reduction in mRNA expression levels at 24 h and 48 h PBM, respectively (Figure 8A), and to a corresponding reduction of 44% in protein levels at 24 h PBM (Figure 8B and C).

**Figure 9:**
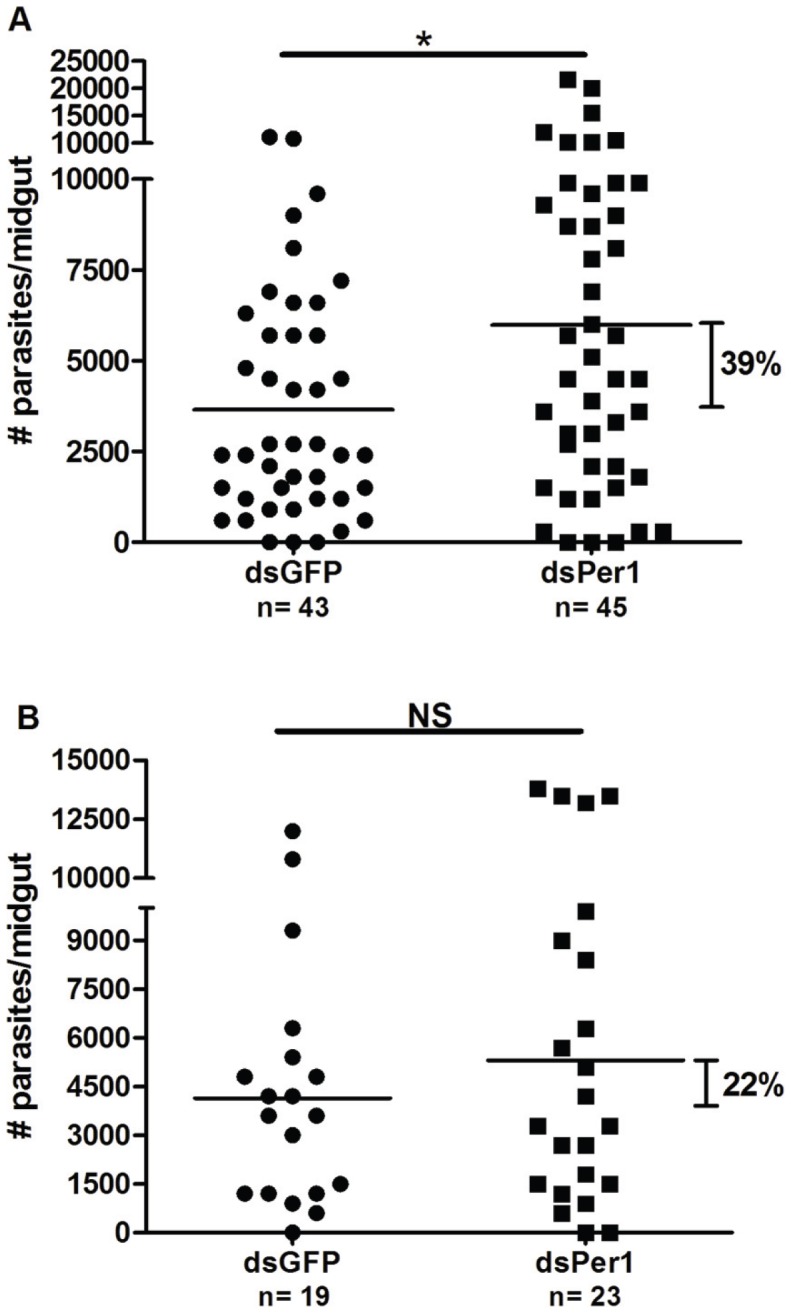
Effects of PpPer1 knockdown on *Le. major* infection. Knockdown of PpPer1 leads to greater *Le. major* load in the midgut of *P. papatasi*. At 48 h post-infection (**A**), dsPpPer1 (dsPer1) injection caused an increased (39%; p<0.05 Mann-Whitney U test) in *Le. major* load compared to dsGFP-injected *P. papatasi*. Results shown are from combined data of two independent experiments. (**B**) Although not statistically significant, *PpPer1* knockdown led to 22% increase in *Le. major* load in *P. papatasi* midguts at 96 h post-infection when compared to dsGFP-injected. Each dot (filled circle or square) represents number of *Le. major* in a single midgut whereas horizontal bars indicate mean parasite number. *P. papatasi* were infected with 5×10^6^ *Le. major* amastigotes/ml in heat-inactivated mouse blood. N: Number of *P. papatasi* midguts dissected. NS: Not significant. *: Statistically significant, p<0.05.

In spite of the lack of noticeable differences in PM structure between dsPpPer1 RNA-injected versus control-injected sand flies following midgut dissection (not shown), knockdown of PpPer1 led to an increase in *Le. major* load within *P. papatasi* midguts of 39% at 48 h and 22% at 96 h post-infection (as shown in Figures 9A and 9B).

## Discussion

Here, we characterized three peritrophins of the sand fly *P. papatasi* thought to be involved in the formation of the PM in adult females. In addition, as the PM is an important component of vector competence in sand flies [7], [16], [17], [30], we assessed the role of PpPer1 as a molecular barrier against *Le. major* development.

Different than PpPer1 and PpPer2, PpPer3 is a mucin-like peritrophin with two CBDs and a mucin-like domain rich in serine, threonine, glutamine, and proline residues. Mucin-like domains are predicted to be heavily *O*-linked glycosylated that contributes to a gel-like consistency for the PM, critical to their role in protecting the midgut epithelia from abrasion, hydrolytic enzymes, heavy metals, and pathogens [38], [39].

In addition to their role in PM formation, peritrophins are also known to participate in detoxification [40]. In the mosquito *A. aegypti*, the mucin-like peritrophin AeIMUC was shown to bind heme *in vitro* via heme-regulatory motifs (HRM), while it also bound chitin [40], [41]. HRMs are predicted for all three *P. papatasi* as well as the *L. longipalpis* peritrophins, suggesting a role for these proteins in heme binding and detoxification in sand flies.

We identified the CBDs present in the three peritrophins from *P. papatasi* and those identified in *L. longipalpis* sequence databases as type-A CBDs displaying the molecular hallmarks required for chitin binding [23], [42], [43]. Chitin binding hallmarks include six conserved cysteine residues (with a conserved number of residues between each conserved cysteines matching the consensus sequence for type-A CBDs) [23], and conserved aromatic amino acids residues predicted to interact with chitin fibrils [23], [43].

Although the two putative CBDs Pp3put and Ll3put display the six conserved cysteines interspaced by the characteristic length expected for type-A CBDs [23], amino acids other than aromatics are found in these putative domains. In addition, the Pp3put and Ll3put CBDs display two extra cysteines at residues 20 and 54 and have an unusual two-peptide insertion between the fourth and fifth conserved cysteines. Such features are in contrast with all the other *bona fide* sand fly type-A CBDs that display a conserved number of amino acids between the fourth and fifth cysteines (12 residues), and lack the extra cysteines present in the putative domains. Thus, it is tempting to speculate that Pp3put and Ll3put CBD domains underwent some type of neo-functionalization [44], and that such change might also have occurred in peritrophins of other insects, such as the cat flea and mosquitoes. This hypothesis is to some extent supported by our findings that PpPer3 (which has the Pp3put domain) also is expressed in the Malpighian tubules, and by the lack of binding of rPpPer3 to chitin (see below). Although the latter may be due to other factors, the expression in tissues other than the sand fly gut is suggestive of an additional function for this protein. Future functional characterization of these putative domains, both in *P. papatasi* and in *L. longipalpis*, will shed light on their functions.

We also obtained recombinant rPpPer1, rPpPer2, and rPpPer3 and confirmed the ability of rPpPer1 and rPpPer2 to bind colloidal chitin and thus likely be involved in the formation of PM1 in *P. papatasi*.

Our assessment of temporal and spatial expression of the *P. papatasi* peritrophins demonstrated that *PpPer1* expression is midgut-specific and blood-induced, resembling the transcriptional profile of *PpChit1*, a midgut specific *P. papatasi* chitinase [32]. The PpPer1 protein appears to be secreted in the midgut at an earlier time point (24 h PBM) following a blood meal than PpChit1, whose activity peaks between 48 h and 72 h PBM [30]. Nonetheless, these patterns of protein expression are consistent with the functional roles of PpPer1 and PpChit1 in PM formation and degradation, respectively.

In contrast to the results observed for *PpPer1*, *PpPer2* and *PpPer3* mRNAs are expressed prior to blood feeding (constitutively), and their expression is not limited to the midgut: PpPer2 is expressed in the midgut and hindgut; while PpPer3 is expressed in the midgut, hindgut, and Malpighian tubules. Although peritrophin expression in hindguts and/or Malpighian tubules have also been detected in the cat flea *Ctenocephalides felis* [45] and the fruit fly *Drosophila melanogaster* [46], the physiological roles of peritrophins in these tissues have not been determined. Regarding the constitutive expression patterns of *PpPer2* and *PpPer3*, the corresponding proteins might not be translated in the same fashion. In *Aedes aegypti*, the peritrophin *AeIMUCI* is constitutively expressed, and yet the protein is only detected in blood fed midguts and up to 24 h PBM [40]. *PpPer2* also is expressed in larval stages, similar to *AeIMUCI* [41]. However, following our assessment of midgut lysates for native peritrophin expression, it became evident that at least for PpPer1, protein expression in the midgut correlates with mRNA expression. Native PpPer1 is readily detected at 48 h PBM, matching its peak of mRNA expression. For native PpPer3, we observed significant levels of protein at 48 h, and lower amounts at 72 h and 96 h PBM. This profile correlates at least partially with the mRNA expression profiles observed.

To assess whether or not *Le. major* infection is capable of modulating *P. papatasi* peritrophin gene expression, we compared *PpPer1*, *PpPer2*, and *PpPer3* mRNA levels between *Le. major* infected and blood fed midguts. Although *Le. major* infection was not able to modulate *PpPer2* expression profile, *PpPer1* and *PpPer3* expression levels changed significantly upon infection. Regulation of peritrophins was suggested by previous transcriptome analyses studies of both *P. papatasi* and *L. longipalpis* [18], [19], [47]. The expression of *PpPer1* was up-regulated at 24 h post-infection whereas *PpPer3* mRNA levels were reduced at 24 h and 48 h post-infection. Up-regulation of *PpPer1* by *Le. major* may assist in protecting the parasite against proteolytic enzymes (parasite advantage), or may be a response by the sand fly in order to possibly reduce permeability of the PM (disadvantageous to the parasite). Whether one or multiple signals secreted by the parasite or present in the infected blood are involved in this regulation still needs to be determined. Similarly, regarding *PpPer3*, whether differential gene expression in infected midguts was parasite-mediated, or a vector-induced defensive response against infection, needs to be further investigated.

The role of the *P. papatasi* peritrophin PpPer1 in *Le. major* development in *P. papatasi* midgut was assessed via RNA interference (RNAi). PpPer1 was chosen for RNAi experiments because it was shown to be expressed exclusively in the midgut, and only after blood feeding.

The sand fly PM is thought to fulfill two apparently opposing roles (protection and barrier) when it comes to *Leishmania* infection [2]. That sand fly PM protects *Le. major* against digestive enzymes early in infection [7] initially suggested to us that a potential alteration of the PM scaffold, increasing its permeability by the knockdown or removal of one or more peritrophins, would lead to killing of parasites. The injection of dsPpPer1 into *P. papatasi* thorax reduced *PpPer1* mRNA and PpPer1 protein levels by 45%. Although no difference in the prevalence of infected sand flies was observed at 48 h post-infection (not shown), PpPer1 knockdown led to a significant increase (39%) in *Le. major* load in *P. papatasi* midguts. One possibility to explain these results is that a PM with increased permeability may allow a greater influx of digestive enzymes to the endoperitrophic space, turning blood meal digestion faster and making nutrients more readily available for *Le. major* multiplication. Although increased *Le. major* load in *PpPer1* knockdown also was noted at 96 h post-infection (22%), this increase was not statistically significant. Our results showing that knockdown of PpPer1 with concomitant increase in parasite load at 48 h post infection do not exactly contradict those of Pimenta et al. [7]. In that case, killing of *Leishmania* was induced by the complete lack of the PM by addition of exogenous chitinases to the blood meal which might also have affected the parasite's own chitinase. In contrast, here, we specifically knocked down a single peritrophin but, overall, the PM was still present likely leading to the distinct outcome between the two studies: killing of *Leishmania* versus increased load. Future studies to assess the changes in PM porosity caused by the knockdown of PpPer1 and how such changes may affect the flow of digestive proteases and nutrients in and out of the endoperitrophic space will help clarify these issues. Recently, Araujo et al [48] showed that adding chitinase to the blood meal led to accelerated egg-laying by sand flies and a reduction of the total eggs laid per females, and it was likely associated with a speedier acquisition of nutrients due to the lack of the PM. Conversely, current data from our laboratory (not shown) indicate that feeding flies with antisera targeting the sand fly midgut chitinase PpChit1 [32] leads to a delay in egg laying after blood feeding and an increase the number of eggs laid per female. Taken together these results suggest that the integrity and the permeability of the PM interfere with the development of *Leishmania* as well as with fitness in sand flies.

## Supporting Information

Figure S1 Fractionation by gel filtration chromatography of *P. papatasi* midgut lysate, and of rPpPer1, rPpPer2, and rPpPer3. Lysate from 10 *P. papatasi* midguts dissected 48 h PBM (native proteins) (A), and rPpPer1 (B), rPpPer2 (C) and rPpPer3 (D) were fractionated using HPLC. The A_280_ absorption spectra for the eluted samples following HPLC fractionation were obtained and 10 µl from each fraction were placed on nitrocellulose membranes and incubated with specific antisera (A and D) or with anti-His antibodies (B and C). Numbers on the A_280_ spectra refer to retention time for each peak. (A) Dot blots containing midgut lysate fractions were incubated with anti-PpPer1 (1) and anti-PpPer3 (2) antisera, respectively. (D) Dot blot was incubated with anti-PpPer3 antisera. Numbers on top of dot blots refer to each fraction collected (fractions were blotted vertically).
(TIFF)
