# Supplementary figures and images for: Characterization of Phlebotomus papatasi Peritrophins, and the Role of PpPer1 in Leishmania major Survival in its Natural Vector
Source: PLoS Negl Trop Dis. 2013 Mar 14;7(3):e2132. doi: 10.1371/journal.pntd.0002132 (PMC3597473; doi:10.1371/journal.pntd.0002132)

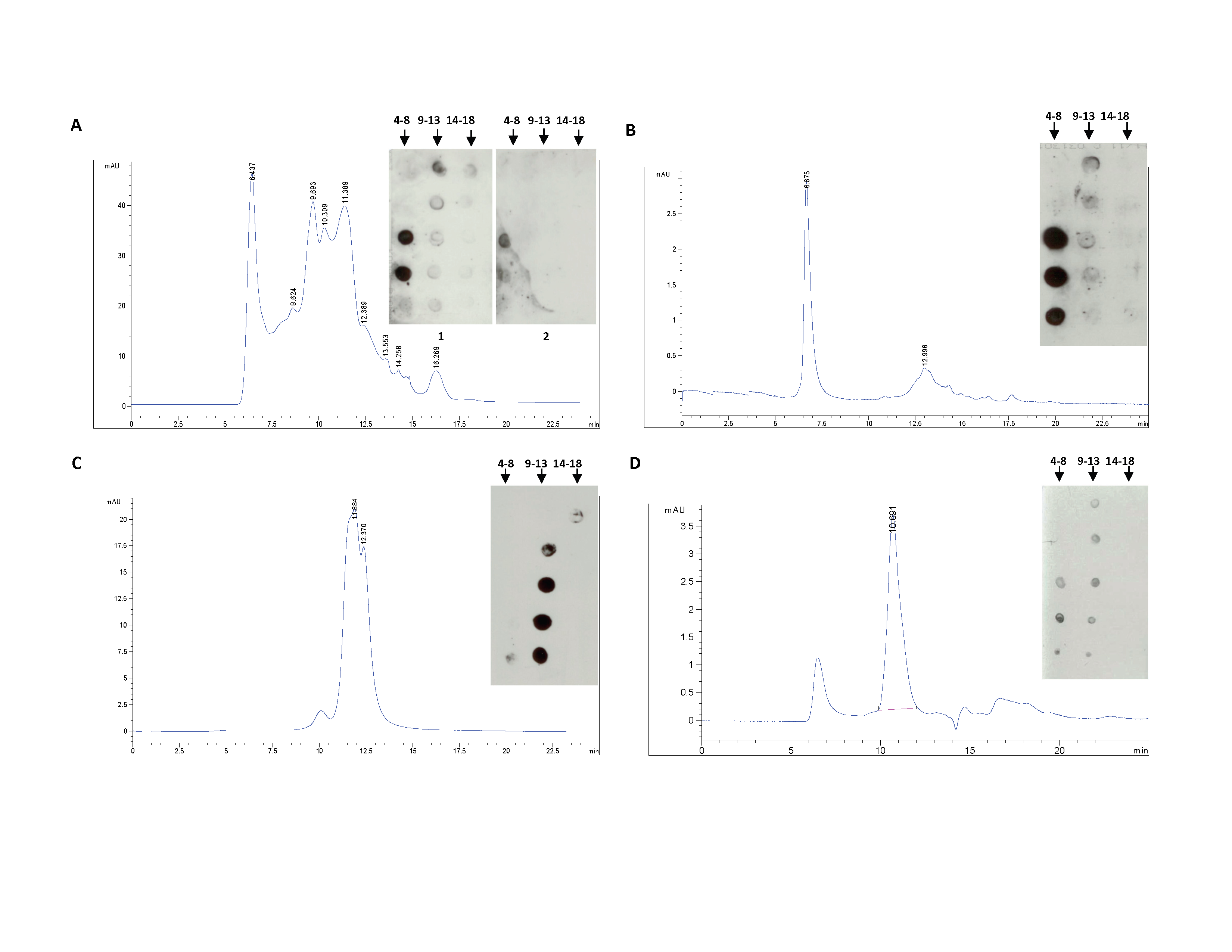

Supplement: Figure S1 — Fractionation by gel filtration chromatography of P. papatasi midgut lysate, and of rPpPer1, rPpPer2, and rPpPer3. Lysate from 10 P. papatasi midguts dissected 48 h PBM (native proteins) (A), and rPpPer1 (B), rPpPer2 (C) and rPpPer3 (D) were fractionated using HPLC. The A280 absorption spectra for the eluted samples following HPLC fractionation were obtained and 10 µl from each fraction were placed on nitrocellulose membranes and incubated with specific antisera (A and D) or with anti-His antibodies (B and C). Numbers on the A280 spectra refer to retention time for each peak. (A) Dot blots containing midgut lysate fractions were incubated with anti-PpPer1 (1) and anti-PpPer3 (2) antisera, respectively. (D) Dot blot was incubated with anti-PpPer3 antisera. Numbers on top of dot blots refer to each fraction collected (fractions were blotted vertically). (TIFF) [file pntd.0002132.s001.tiff]
